# From Resistance Mechanism to Green Application: Discovery of Rutaevin as a Key Phytoalexin in Larch and Cross-Species Resource Optimization

**DOI:** 10.3390/plants14192947

**Published:** 2025-09-23

**Authors:** Ruizhi Zhang, Shuang Zhang, Rui Xia, Xinyan Chen, Jiarui Chen, Feng Wang, Danlei Li

**Affiliations:** 1 Key Laboratory of Alien Forest Pest Detection and Control-Heilongjiang Province, College of Forestry, Northeast Forestry University, Harbin 150040, China; zhangruizhi@nefu.edu.cn (R.Z.); zhangshuang1666@163.com (S.Z.); xiarui@nefu.edu.cn (R.X.); cxy0201@nefu.edu.cn (X.C.); chenjr220924@163.com (J.C.); 2 Key Laboratory of Sustainable Forest Ecosystem Management-Ministry of Education, College of Forestry, Northeast Forestry University, Harbin 150040, China; 3 State Key Laboratory of Tree Genetics and Breeding, College of Forestry, Northeast Forestry University, Harbin 150040, China

**Keywords:** rutaevin, phytoalexin, *Neofusicoccum laricinum*, larch, antifungal, *Evodia rutaecarpa* var. *rutaecarpa*

## Abstract

*Neofusicoccum laricinum*, the pathogen responsible for larch shoot blight, is a hemibiotrophic pathogen. A hypersensitive reaction of plants does not inhibit the growth of the pathogen, while phytoalexin is an antifungal compound secreted by plants, which can directly destroy the cells of pathogens and help plants achieve resistance. This study aimed to investigate the chemical defense mechanisms in resistant larch, identify key biocontrol agents, and assess their potential for field application. By integrating multi-omics technologies with time-dependent models and dose–response curve analysis, the accumulation and antimicrobial properties of rutaevin were examined. Its application potential was verified through cross-species resource screening and field trials. The results revealed that rutaevin acts as a phytoalexin against larch shoot blight. It began to accumulate linearly 0.62 days after pathogen exposure, with its antifungal activity demonstrating a dose-dependent response, achieving 100% inhibition at 0.5 mg/mL. The activation of terpene metabolic pathways in disease-resistant plants resulted in a significant increase in rutaevin content compared to susceptible plants. Cross-species screening showed that the highest concentration of this compound is found in the fruit of *Evodia rutaecarpa* var. *rutaecarpa*, with its crude extract exhibiting strong field efficacy. The findings provide theoretical and technical support for disease-resistant breeding and the development of plant-derived fungicides.

## 1. Introduction

Larch (*Larix* spp.) forests serve as key components of forest ecosystems in Northeast Asia and Europe, playing an essential role in maintaining global climate and ecological security [1,2]. Larch shoot blight, caused by the invasive pathogen *Neofusicoccum laricinum* [3,4], has posed a serious threat to forests in Northeast Asian countries such as Russia, North Korea, and China [5,6,7]. Additionally, larch lacks specific resistance mechanisms against this pathogen [6]. The absence of heterologous resistance mechanisms in larch outside Japan has facilitated the outbreak and spread of larch shoot blight [8]. Since the disease was first identified in the 1970s, it has affected over 667,000 hectares of forest, causing severe damage to larch resources and forest ecosystems [9,10,11]. However, the current disease control system has significant deficiencies in terms of timeliness, ecological safety, and sustainability. Effective control of larch shoot blight outbreaks and their spread, particularly through the development of environmentally friendly and efficient biocontrol fungicides, remains an urgent challenge [8]. Due to its complex life history, there are difficulties in the prevention and control of larch shoot blight. At present, the most effective prevention and control measures are chemical control and appropriate forest management measures [8]. However, chemical control is only effective in a short period of time and will cause huge environmental pollution. Larch shoot blight is a host-dominant disease. Breeding disease-resistant larch and finding biological control methods based on disease-resistant larch will provide new measures for the prevention and control of larch shoot blight. To overcome the limitations of traditional control methods, biological control strategies based on plant immune mechanisms have attracted attention, with chemical defense related to phytoalexins being a key focus.

*N. laricinum* is a hemibiotrophic pathogen. An effective strategy for plants to respond to hemibiotrophic pathogens involves the synthesis of phytoalexins, a class of antifungal secondary metabolites induced during pathogen infection [12,13,14,15]. Phytoalexins serve as a core defense mechanism against hemibiotrophic pathogens, as they suppress the growth and reproduction of the pathogen in a dose-dependent manner during both the biotrophic and necrotrophic stages of the pathogen’s life cycle [16,17]. Our previous research found that, despite the lack of a co-evolutionary history between native Chinese larch and *N. laricinum*, approximately 5% to 10% of larches in nature exhibit disease resistance [6,18,19]. These resistant larches possess different disease resistance mechanisms, but all exhibit antifungal activity through the synthesis of broad-spectrum antifungal phytoalexins such as vanillic acid, farrerol, and vanillin [6]. These broad-spectrum antifungal phytoalexins confer non-specific resistance by damaging conserved targets like the pathogen’s cell membrane [20,21,22]. The diversity of broad-spectrum antifungal phytoalexins in different resistant larch varieties provides a breakthrough for controlling invasive pathogens and represents a valuable resource for the development of plant-derived fungicides.

In this study, rutaevin was identified through metabolomics analysis from the disease-resistant *Larix olgensis*, which serves as a phytoalexin capable of inhibiting the growth of *N. laricinum*. *Evodia rutaecarpa* (Rutaceae family) is a widely used traditional Chinese medicinal herb traditionally prescribed for gastrointestinal issues and headaches. This plant is rich in metabolites such as evodol, shihulimonin A1, evodirutaenin, 12α-hydroxyrutaevin, and rutaevin, all of which are structurally related [23,24]. Recently, limonoids isolated from the fruit of *E. rutaecarpa* have gained significant attention due to their neuroprotective potential [25,26]. Among these, rutaevin, a major limonoid, has demonstrated cytotoxic effects against NCI-N87 and Caco-2 cell lines. Other studies have also shown that oral administration of rutaevin can lead to hepatotoxicity in mice, with bioactivation processes believed to play a role in this liver damage [27]. Currently, limited research exists on the role of rutaevin in plant disease resistance, and studies on its inhibitory effects against plant pathogen and the underlying mechanisms are scarce. Further investigation into the antifungal properties of rutaevin and its development as a plant-derived fungicide could provide new research directions for controlling larch shoot blight.

This study aimed to reveal the molecular mechanisms of disease resistance in larch against *N. laricinum* and to screen for phytoalexins suitable for use in control trials. By screening the phytoalexins in disease-resistant larch, rutaevin was identified. Further experiments confirmed that these metabolites exhibited significant antifungal activity against *N. laricinum*. Various *Evodia* fruit extracts were screened, and superior plants producing high yields of rutaevin were selected. Small-scale control trials demonstrated the effectiveness of the extracts, providing technical support for large-scale applications. This study revealed the molecular mechanisms by which disease-resistant larch enhances secondary metabolic pathways to respond to pathogen infection, particularly emphasizing the key role of broad-spectrum antifungal phytoalexins. It provides new insights into plant disease resistance mechanisms and offers theoretical and technical support for the development of plant-derived fungicides.

## 2. Results

### 2.1. Discovery of Rutaevin as a Critical Disease-Resistant Metabolite in Larch

**Figure 1:**
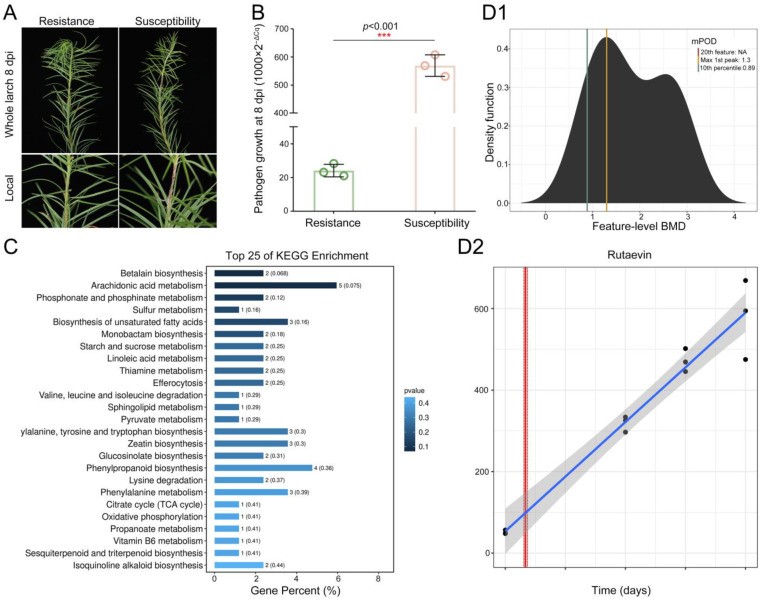
Time-dependent accumulation of rutaevin in *L. olgensis* during resistance to larch shoot blight. (**A**) Phenotypic comparison of *L. olgensis* after *N. laricinum* inoculation. Resistant plants show minimal symptoms, while susceptible plants exhibit browning and necrosis. (**B**) Pathogen biomass quantification by qPCR shows a 95.75% reduction in pathogen growth in resistant larches compared to susceptible ones (*p* < 0.001). (**C**) Metabolomic profiling identifies 204 significantly upregulated metabolites in resistant *L. olgensis* (fold change > 1.2, *p* < 0.05) enriched in phytoalexin-related pathways. (**D**) Rutaevin accumulation over time. (**D1**) Accumulation of the 204 metabolites reveals 5 metabolites’ time-dependent increase in resistant *L. olgensis*. (**D2**) Rutaevin shows a linear accumulation pattern over time, with a significant increase after 0.62 days of pathogen exposure (lack-of-fit test: *p* = 0.81).

To elucidate larch’s chemical defense against shoot blight caused by *N. laricinum*, we integrated phenotypic and multi-omics analyses. Resistant plants showed no cell death (Figure 1A), while susceptible plants exhibited browning/necrosis. Pathogen growth was reduced by 95.75% in resistant larch (*t*(4) = −24.38, *p* < 0.001, Cohen’s *d* = −19.91, 95% CI [−39.58, −0.23], Figure 1B).

Integrated metabolomics analyses demonstrated significantly higher levels of 204 metabolites in resistant larch (fold change > 1.2, *p* < 0.05, Figure S1) enriched in phytoalexin synthesis pathways including phenylpropanoid biosynthesis, sesquiterpenoid and triterpenoid biosynthesis, glucosinolate biosynthesis, isoquinoline alkaloid biosynthesis, and sulfur metabolism. Rutaevin (a limonoid) is associated with citrate cycle and sesquiterpenoid/triterpenoid biosynthesis (Figure 1C).

Time–response modeling of these metabolites revealed five fitting predictive trends (Figure 1D1). Only rutaevin showed continuous linear accumulation post-inoculation (linear model; lack-of-fit *p* = 0.81), with significant increases initiating at 0.62 days and peaking by 0.73 days (Figure 1D2). Thus, rutaevin has been established as a key phytoalexin in larch defense through integrated evidence.

### 2.2. Functional Validation of Rutaevin in Larch Defense: Temporal Accumulation Dynamics and Dose-Dependent Antifungal Activity

**Figure 2:**
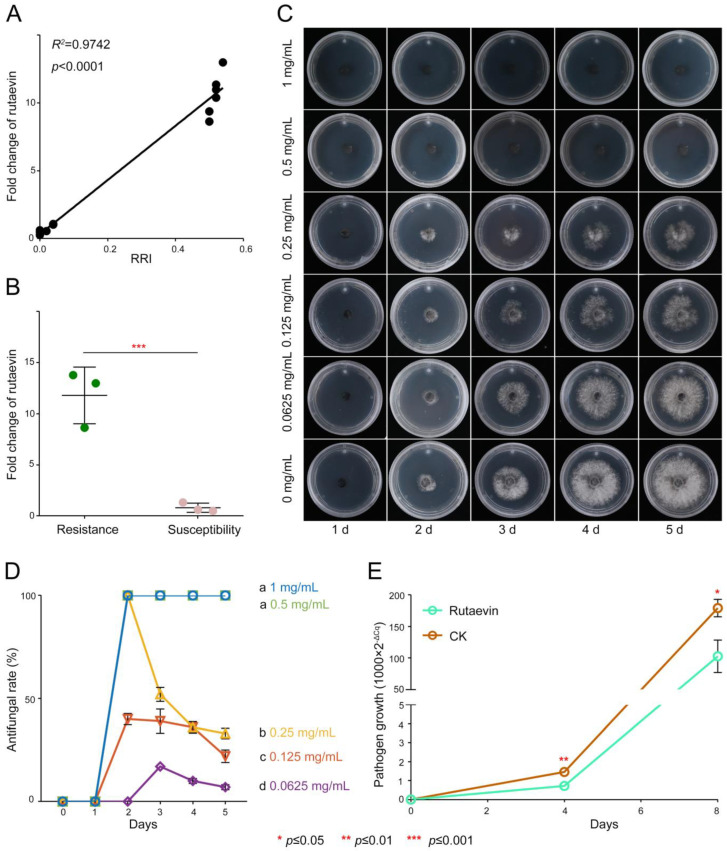
Antifungal activity of rutaevin against *N. laricinum*. (**A**) Correlation between rutaevin content and disease resistance. Resistant *L. olgensis* contains significantly higher rutaevin levels than susceptible *L. olgensis*. A strong positive correlation is observed between rutaevin content and disease resistance. (**B**) Rutaevin accumulation after infection. Following *N. laricinum* inoculation, rutaevin levels increase significantly in resistant larch (fold change = 11.81, *p* < 0.01), but not in susceptible plants. (**C**) Dose-dependent antifungal effect of rutaevin. Antifungal assays show a dose-dependent inhibition of *N. laricinum* by rutaevin, with greater inhibition at higher concentrations. (**D**) Complete inhibition of *N. laricinum* growth. Rutaevin achieves 100% inhibition of *N. laricinum* at 0.5 mg/mL in antifungal assays. Letter a: the maximum average number marked with the letter a. Letter b: The maximum average is compared with the following averages. Where the difference is not significant, the letter a is marked until a significant difference is marked with the letter b. Letter labeling followed by analogy. Where there is an identically marked letter, the difference is not significant; where there are different marked letters, the difference is significant. (**E**) Pathogen biomass reduction. qPCR analysis shows a significant decrease in mycelial DNA in rutaevin-treated (0.25 mg/mL) larches compared to controls (*p* < 0.001).

Resistant larches (51.18 ± 3.90 μg/g) had >40% higher rutaevin than susceptible plants (36.00 ± 3.47 μg/g), correlating strongly with disease resistance indices (52.00 ± 2% vs. 2.67 ± 1.16%; *R*^2^ = 0.9742, *p* < 0.0001; Figure 2A).

Targeted metabolomics confirmed this disparity (fold change = 1.42, *t*(4) = 4.03, *p* = 0.015, Cohen’s *d* = 3.29, 95% CI [−0.65, 7.23], Figure S2) and showed pathogen-induced rutaevin accumulation exclusively in resistant larch (fold change = 11.81, *t*(4) = 6.78, *p* < 0.01, Cohen’s *d* = 5.53, 95% CI [−0.35, 11.42], Figure 2B).

Antifungal assays confirmed a dose-dependent inhibitory effect of rutaevin on *N. laricinum* growth (Figure 2C, Table S1), with 100% inhibition achieved at 0.5 mg/mL (Figure 2D) and a half-maximal inhibitory concentration (IC_50_) value of 0.27 mg/mL (95% CI [0.1951, 0.3449]). Quantitative real-time PCR (qPCR) quantification further confirmed a significant reduction in *N. laricinum* biomass upon rutaevin treatment compared to the dimethyl sulfoxide (DMSO) control (*p* < 0.01; Figure 2E).

Collectively, rutaevin exhibits pathogen-triggered time-dependent accumulation and potent antifungal activity, mechanistically underpinning larch resistance.

### 2.3. Identification of Genes Related to Rutaevin Metabolic Pathway and Derivation of Its Synthesis Mechanism

To explore the molecular mechanism behind rutaevin synthesis in pathogen-induced larch, transcriptomic, proteomic, and non-targeted metabolomic technologies were employed to systematically analyze disease-resistant and susceptible larch samples at 8 days post inoculation (dpi).

Transcriptomic analysis revealed differentially expressed genes (DEGs) associated with secondary metabolism, which were upregulated and primarily enriched in the biosynthesis of terpenoids, indicating their involvement in the synthesis of secondary metabolites and plant protectants (Figure S3). Proteomic analysis confirmed the transcriptomic findings, showing the upregulation of key enzymes (e.g., 3-hydroxy-3-methylglutaryl-CoA reductase (HMGR), 1-Deoxy-D-xylulose-5-phosphate synthase (DXS), Deoxyxylulose 5-phosphate reductoisomerase (DXR), and geranylgeranyl diphosphate synthase (GGDPS)), suggesting the activation of metabolic pathways, likely promoting the synthesis of terpenoids, phenylpropanoids, and other secondary metabolites (Figure S4). Metabolomic analysis showed that the content of secondary metabolites, including rutaevin, was significantly higher in disease-resistant larch compared to susceptible larch. This accumulation of metabolites was consistent with the transcriptomic and proteomic results. The upregulation of secondary metabolism-related genes in disease-resistant larch facilitated the accumulation of various secondary metabolites, with the significant increase in rutaevin closely linked to its disease resistance.

**Figure 3:**
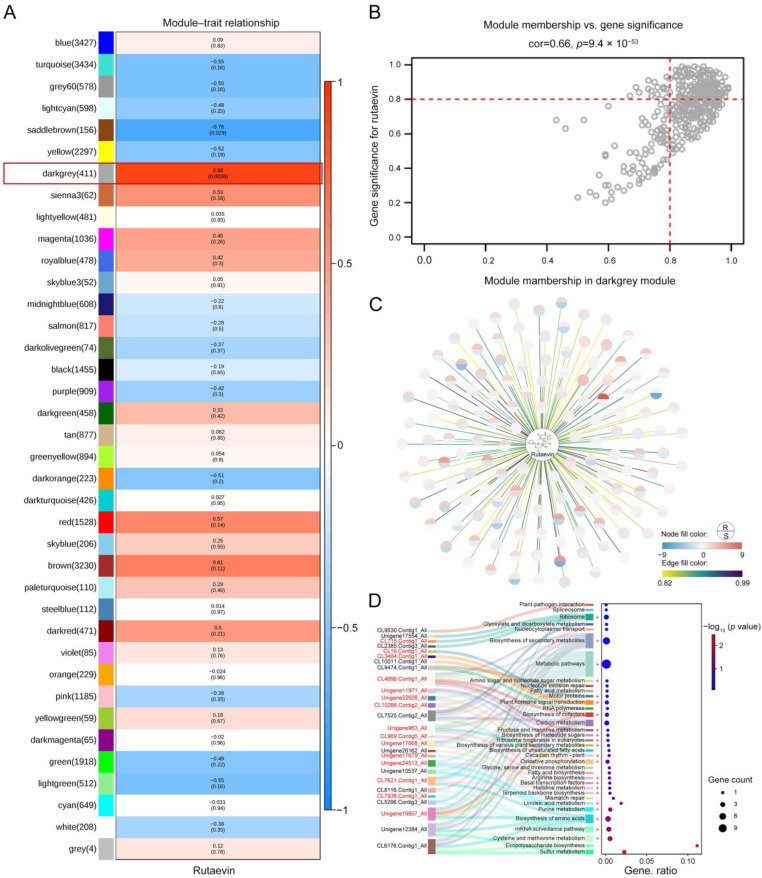
Integration of transcriptomic, proteomic, and metabolomic data identifies key gene modules and pathways associated with disease resistance and rutaevin content. (**A**) WGCNA identified 38 gene modules, with the darkgrey module significantly correlated with changes in rutaevin content (correlation = 0.88, *p* = 0.0039). (**B**) The darkgrey module, comprising 411 genes, showed a strong correlation between MM and GS for rutaevin. (**C**) A subset of 137 genes, with both GS and MM values greater than 0.8, exhibited gene expression trends consistent with changes in rutaevin content. (**D**) KEGG enrichment analysis of the darkgrey module identified 36 enriched pathways across 5 major categories and 15 subcategories. Proteomic data confirmed the transcriptomic results, showing consistent expression patterns for metabolism-related genes and enzymes (marked in red+).

Weighted correlation network analysis (WGCNA) was used to integrate transcriptomic and metabolomic data to construct a gene co-expression network and identify several gene modules significantly associated with disease resistance. A total of 38 different gene co-expression modules were identified through weighted gene co-expression network analysis, with the darkgrey module being significantly associated with changes in rutaevin content (correlation coefficient: 0.88, *p* = 0.0039) (Figure 3A). The darkgrey module contained 411 genes, and the correlation between these genes and the module (Module Membership, MM) as well as their correlation with rutaevin (Gene Significance for rutaevin, GS for rutaevin), was analyzed (Figure 3B). The results revealed a high correlation between GS and MM, indicating that genes highly correlated with rutaevin are often the most important genes in modules related to the trait. A total of 137 genes with both a GS and an MM greater than 0.8 were selected for analysis (Figure 3C). Among these, 62 genes were significantly upregulated in disease-resistant larch, while they remained unchanged or were significantly downregulated in disease-sensitive larch. The gene expression trends were consistent with the changes in rutaevin content. Kyoto Encyclopedia of Genes and Genomes (KEGG) enrichment analysis revealed 36 pathways, grouped under 5 major categories and 15 subcategories, primarily enriched in lipid metabolism (ko00591, ko00061, and ko01040), amino acid metabolism (ko00270, ko00340, ko00220, and ko00260), carbohydrate metabolism (ko00051, ko00520, and ko00630), and terpenoid and polyketide metabolism (ko00900) (Figure 3D). Genes in the darkgrey module were mainly enriched in pathways related to terpenoid biosynthesis, phenylpropanoid biosynthesis, and lignin biosynthesis. Proteomic analysis indicated that the expression levels of metabolism-related genes and enzymes were consistent with the transcriptomic results (Figure 3D, marked in red+). The integration of transcriptomic, metabolomic, and proteomic data using a PLS regression model revealed that the expression levels of secondary-metabolism-related genes significantly predicted the accumulation of rutaevin (*R*^2^ = 0.85, Q^2^ = 0.78, VIP > 1.0), suggesting that its synthesis is regulated by the mevalonate (MVA) and methylerythritol phosphate (MEP) pathway and may be completed through triterpene skeleton modifications mediated by oxidosqualene cyclase (OSC).

In conclusion, plant disease resistance is primarily enhanced through the synthesis and accumulation of secondary metabolites, particularly terpenoids, phenylpropanoids, and glycosides. Disease-resistant larch accumulates rutaevin and other plant protectants by activating secondary metabolic pathways. The antifungal, antioxidant, and systemic acquired resistance (SAR) induction functions of rutaevin are closely related to disease resistance.

### 2.4. Distribution Patterns of Rutaevin Across Different Provenances and Plant Organs: Identification of Optimal Sources for Efficient Extraction and as an Effective Biocontrol Agent Against N. laricinum in Larch

Due to the low rutaevin content (51.18 μg/g) and cultivation challenges in larch, alternative sources like *E. rutaecarpa* (easier to cultivate and extract) were evaluated for rutaevin yield and efficacy against larch shoot blight. We quantified rutaevin content in *E. rutaecarpa* var. *rutaecarpa*, *E. rutaecarpa*, and *E. rutaecarpa* var. *bodinieri* fruits and evaluated their methanol extracts’ antifungal activity against *N. laricinum* and control efficacy on larch shoot blight.

**Figure 4:**
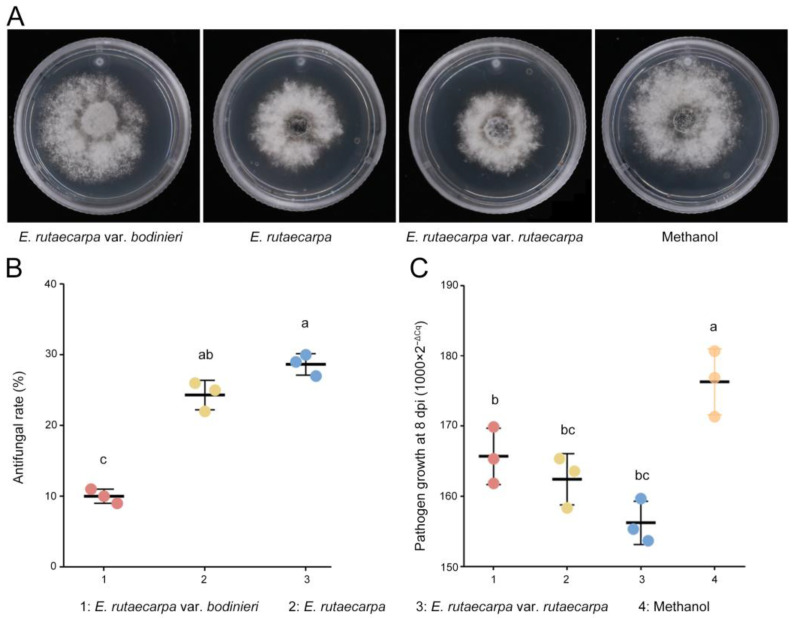
Antifungal activity and disease control efficacy of *E. rutaecarpa* methanol extracts against *N. laricinum* and larch shoot blight. (**A**) Antifungal activity of methanol extracts from 3 *E. rutaecarpa* species. (**B**) Comparison of antifungal efficacy between the 3 *E. rutaecarpa* species. (**C**) Effect of methanol extracts on larch shoot blight. Letter a: the maximum average number marked with the letter a. Letter b: The maximum average is compared with the following averages. Where the difference is not significant, the letter a is marked until a significant difference is marked with the letter b. Letter labeling followed by analogy. Where there is an identically marked letter, the difference is not significant; where there are different marked letters, the difference is significant.

The antifungal assays revealed that methanol extracts from all three *Evodia* species exhibited antifungal activity (Figure 4A). The methanol extract of *E. rutaecarpa* var. *rutaecarpa* displayed the highest antifungal activity (Figure 4B), indicating superior effectiveness.

Further tests on the application of the three methanol extracts to inoculated larch branches showed that the *E. rutaecarpa* var. *rutaecarpa* extract resulted in the lowest fungal mycelium count on diseased larch shoots (Figure 4C), demonstrating the best control efficacy against larch shoot blight. The disease incidence in the treated larch with *E. rutaecarpa* var. *rutaecarpa* extract was 78.33%, which is significantly lower than that in the control group (96.67%, Fisher’s exact test, *p* = 0.029). The 95% confidence interval for disease incidence was [60.87%, 89.36%] for the *E. rutaecarpa* var. *rutaecarpa* extract group and [83.33%, 99.41%] for the control group. The DI was also significantly reduced to 31.25%, lower than the untreated group (43.75%, *p* = 0.048). The 95% confidence interval for DI was [17.62%, 49.14%] for the *E. rutaecarpa* var. *rutaecarpa* extract group and [27.73%, 61.19%] for the control group. These results indicate that the treatment significantly reduced the incidence of larch shoot blight.

**Figure 5:**
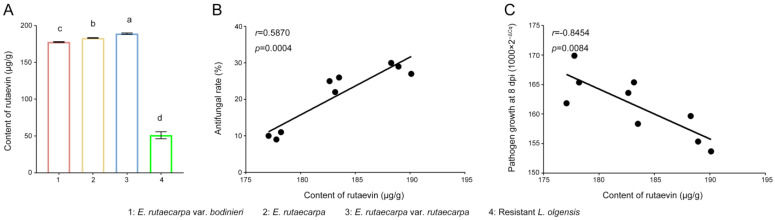
Rutaevin content and its correlation with antifungal activity in *E. rutaecarpa* species. (**A**) Quantification of rutaevin content in fruits of *E. rutaecarpa* species and larch. (**B**) Correlation between rutaevin content and mycelial growth inhibition. (**C**) Correlation between rutaevin content and the control of larch shoot blight. Letter a: the maximum average number marked with the letter a. Letter b: The maximum average is compared with the following averages. Where the difference is not significant, the letter a is marked until a significant difference is marked with the letter b. Letter labeling followed by analogy. Where there is an identically marked letter, the difference is not significant; where there are different marked letters, the difference is significant.

Targeted metabolomics analysis was employed to quantify the rutaevin content. Quantitative analysis (Figure 5A) showed that the rutaevin content in *E. rutaecarpa* var. *rutaecarpa* fruits (183.11 μg/g) was 3.58-fold higher than in larch (51.18 μg/g; *p* < 0.01). The variation in rutaevin content within each *E. rutaecarpa* fruit group was low (SD = 4.69 μg/g, CV = 2.56%), indicating stable rutaevin content across species. Significant differences in rutaevin content were found among the three Evodia species (F = 211.775, *p* < 0.001), with *E. rutaecarpa* var. *rutaecarpa* exhibiting the highest levels (Figure 5A).

The extraction efficiency of rutaevin from three *E. rutaecarpa* varieties showed a significant positive correlation with the antifungal effect of methanol extracts on *N. laricinum* growth (*r* = 0.5870, *p* = 0.0004, Figure 5B), but a strong negative correlation with pathogen growth on *N. laricinum*-infected larch shoots (*r* = −0.8454, *p* = 0.0084, Figure 5C). These results indicate that rutaevin is the primary bioactive compound in methanol extracts responsible for the observed antifungal activity.

These findings demonstrate that *E. rutaecarpa* var. *rutaecarpa* fruits are an ideal source for rutaevin extraction, with environmental conditions and genetic factors likely influencing its yield. Rich in plant-derived compounds like rutaevin, these fruits exhibit potent bioactivity for disease control, particularly against larch shoot blight.

## 3. Discussion

The outbreak of larch shoot blight, caused by *N. laricinum*, presents a significant threat to the ecological security of Northeast Asia, highlighting the urgent need for the development of effective and environmentally friendly plant-derived fungicides for disease management. This study identifies that disease-resistant larch can synthesize rutaevin, a plant-derived fungicide. Additionally, fruits from *E. rutaecarpa* var. *rutaecarpa* were identified as an excellent source for rutaevin extraction, which can be utilized for controlling larch shoot blight. These findings establish a foundation for the cross-species use of this compound and provide significant theoretical and technical support for research into larch disease resistance mechanisms and the development of plant-derived fungicides.

This study systematically reveals the central role of rutaevin in the disease resistance mechanism of larch, with important implications for innovation and application. In terms of scientific discovery, a multi-omics integration approach was employed to construct a “phenotype–metabolite–gene module” evidence chain, confirming the disease-resistant function of rutaevin in coniferous species. Through a time-dependent accumulation model and dose–effect curve, dynamic defense characteristics were quantitatively analyzed, providing precise data on the disease resistance timeline. In terms of mechanism analysis, a novel combination of PLS regression and WGCNA networks was used to identify key gene modules, such as *HMGR* and *DXS*, in the *MVA*/*MEP* pathway. It was revealed that rutaevin forms an integrated disease resistance network through three defense mechanisms: direct antimicrobial action, reactive oxygen species (ROS) scavenging, and enhanced lignin deposition. Finally, in terms of application, it was found that the rutaevin content in *Evodia* fruits was more than 3.58 times higher than that in larch.

Field trials of the crude extract significantly reduced disease incidence in susceptible plants from 43.75% to 31.25%, demonstrating the success of “cross-species resource optimization” and providing a new pathway for the development of plant-derived fungicides. In contrast to traditional plant protection research, this study presents a comprehensive innovation through the “target discovery–mechanism analysis–resource screening” approach. This not only provides molecular markers for coniferous tree disease resistance breeding but also establishes a complete technical paradigm, from natural product exploration to green pesticide development. These advancements have significant ecological value, contributing to the green control of forest diseases and reducing dependence on chemical pesticides.

The infection of hemibiotrophic pathogen triggers complex defense responses in host plants, involving the salicylic acid (SA) signaling pathway, plant protection compound accumulation, and the synthesis of secondary metabolites [28,29,30,31]. The accumulation of secondary metabolites is a critical strategy for host plants to respond to pathogen infection, with the synthesis of phytoalexins playing a key role in inhibiting pathogen growth [32,33,34,35]. Multi-omics integration revealed the molecular mechanisms of disease-resistant larch in response to *N. laricinum* infection, showing that its defense largely depends on the synthesis and accumulation of secondary metabolites to enhance plant disease resistance. These secondary metabolites include terpenes, phenylpropanoids, and glycosides. Specifically, genes such as *HMGR* and *DXS*/*DXR* are involved in the MVA and MEP pathways, while *GPPS* and *OSC* genes promote the generation and modification of triterpene metabolites. Andrographolide and phorbol esters enhance disease resistance by inhibiting pathogen growth and interfering with the infection process [36]. Moreover, UDP-glycosyltransferases (*UGT*) genes play a key role in the biosynthetic pathway of glycosides, enhancing their water solubility and stability, and thus increasing their toxicity to pathogens [37,38].

These secondary metabolites significantly improve larch’s disease resistance through mechanisms such as directly inhibiting pathogen growth, enhancing immune responses, improving cell wall structure, scavenging ROS, alleviating oxidative stress, and regulating [32,39,40,41]. This study not only details the secondary metabolic pathways in disease-resistant larch but also clarifies the functions of the relevant genes and their roles in the disease resistance response. These findings provide important theoretical support and application prospects for the development of efficient, environmentally friendly plant-derived fungicides. Moreover, the results align with research on disease resistance mechanisms in other plants, further enriching the understanding of plant–pathogen interactions at the molecular level.

While the key role of rutaevin in larch’s resistance to larch shoot blight and its synthesis regulatory network have been preliminarily revealed, several limitations remain. First, although key gene modules in the *MVA*/*MEP* pathways, such as *HMGR* and *DXS*, were identified through multi-omics integration, the upstream transcriptional regulators (e.g., *MYB* or *NAC* families) and epigenetic modifications regulating rutaevin synthesis remain unclear. Future studies may combine single-cell sequencing with CRISPR/Cas9 gene-editing technology to target the knockout of candidate regulatory genes (e.g., *OSC* family members), clarifying their spatiotemporal-specific contributions to rutaevin synthesis and analyzing their interactions with jasmonic acid/SA signaling pathways. Second, although the field efficacy of crude extracts from *Evodia* fruits was significant, further optimization is required to address the stability of the active compounds, the scalability of the extraction process, and environmental adaptability. Further development of a rutaevin nano-delivery system is necessary to assess its sustained-release performance and antifungal spectrum expansion potential in complex field environments. Additionally, exploring its ecological compatibility in combination with probiotics for disease management could help reduce dependency on single compounds. It is noteworthy that although this study focuses on coniferous species, the “secondary metabolism–defense network” regulatory paradigm revealed here may be applicable to other forest disease systems [42]. For example, the specific inhibition mechanism of rutaevin against hemibiotrophic pathogen may offer new strategies for controlling cross-species compound infection. Future studies can further verify its cross-species defense functions in broadleaf species or crops, promoting the universal development of plant protection compounds for cross-species use.

Through multi-omics analysis, the molecular defense mechanisms of disease-resistant larch against *N. laricinum* infection were systematically elucidated. This study revealed the key role of rutaevin in disease resistance. By screening extracts from different *Evodia* fruits, high-yielding rutaevin plants were identified, and a small-scale control test showed control efficacy, significantly enhancing the potential of this plant protection compound for practical application. The findings not only provide a new perspective for research on plant disease resistance mechanisms but also lay the theoretical and technical foundation for the development of efficient and environmentally friendly plant-derived fungicides. Additionally, *E. rutaecarpa* var. *rutaecarpa* fruits, as an efficient source of rutaevin extraction, offer an important reference for future large-scale development and application. This study has significant implications for the green control of larch shoot blight and sustainable forestry development, while also providing new strategies and technical support for the management of other plant diseases.

## 4. Materials and Methods

### 4.1. Materials

Disease-resistant and susceptible *L. olgensis*, *E. rutaecarpa* var. *bodinieri*, *E. rutaecarpa*, and *E. rutaecarpa* var. *rutaecarpa* fruits were obtained from the Key Laboratory of Alien Forest Pest Detection and Control, Northeast Forestry University, Heilongjiang Province. The *N. laricinum* HLJ001 strain was isolated from diseased *L. olgensis* in Shangzhi City, Heilongjiang Province, and identified through morphological and molecular biological methods [6].

### 4.2. Artificial Inoculation of L. olgensis with N. laricinum

The HLJ001 strain was inoculated onto one-year-old branches of both resistant and susceptible *L. olgensis* to assess phenotypic differences and pathogen dynamics following *N. laricinum* infection. Three replicates were performed for each plant. A ddH_2_O mock inoculation was used as a control. Inoculated branches were incubated in a growth chamber (25 °C, 80–90% relative humidity, 16 h of light, 8 h of darkness) and monitored until symptoms of larch shoot blight appeared. Symptoms were documented photographically (Canon, EOS 6D) for further analysis [18].

Infected branches were classified into the following grades:

**Grade 0:** healthy;

**Grade I:** stem de-greened, few needles shed;

**Grade II:** stem yellow-brown, approximately 50% needle loss, shoot tip slightly drooping;

**Grade III:** stem brown, most needles shed, shoot tip drooping;

**Grade IV:** stem dark brown, all needles shed except for a cluster of purple-gray necrotic needles at the tip.

The disease index (DI) was calculated, followed by the relative resistance index (RRI). A *t*-test was used to assess resistance differences between the resistant *L. olgensis* and the susceptible *L. olgensis* [6].

(1) $$DI=\left[\frac{\left(0n_{0}+1n_{I}+2n_{II}+3n_{III}+4n_{IV}\right)}{4n}\right]\times 100$$

(2) $$RRI=1-\left[\frac{DIx}{DIy}\right]$$

In these formulas, the number of plants at each disease grade (n_0_–n_IV_) and the total number of plants surveyed (*n*) were used to calculate *DI*. The *DI* for each plant (*DI_x_*) and the highest *DI* (*DI_y_*) were used to compute the RRI.

Quantification of *N. laricinum* biomass in resistant and susceptible *L. olgensis* was performed using qPCR (Promega, Madison, Wisconsin, USA; directory number, A6010), employing species-specific primers for the ITS regions of *L. olgensis* and *N. laricinum* (primer sequences, *Larix*-ITS-F: 5′- CTT TGT TGA TGG GTG CCA AT -3′ and *Larix*-ITS-R: 5′- GGA AAT CTC GAG GCA AGA AGA -3′; *NL*-ITS-F: 5′- CTT GTT TCT CAG ACT GCG ACG -3′ and *NL*-ITS-R: 5′- CTC GAC TCT CCC ACC CCA T -3′) [43,44]. The calculation formula is as follows:

(3) $$pathogen\;growth=1000\times 2^{-∆Cq}$$

### 4.3. Screening of Differential Phytoalexins Using Metabolomics

Non-targeted metabolomics was employed to screen for disease-resistance-related secondary metabolites, while targeted metabolomics (HPLC) was used to quantify rutaevin content.

Samples of *L. olgensis* tissue (1 g) were flash-frozen in liquid nitrogen and stored at –80 °C. Metabolomic analysis was performed by the Beijing Genomics Institute (BGI) using LC-MS/MS. The data were processed using Compound Discoverer for peak extraction and metabolite identification, followed by quality control using metaX [45]. Targeted HPLC analysis quantified rutaevin content in *L. olgensis*, *E. rutaecarpa* var. *bodinieri*, *E. rutaecarpa*, and *E. rutaecarpa* var. *rutaecarpa* fruits by the Suzhou Michy Biomedical Technology Co., Ltd., Suzhou, China. According to the targeted quantification of rutaevin, the purity of rutaevin was ≥98% (source leaf), the purity of methanol was ≥99.9% (OCEANPAK), and the purity of acetonitrile was ≥99.9% (OCEANPAK). An Agilent1100 high-performance liquid chromatograph (wavelength of 210 nm) and a Compass C18 (2) reversed-phase column (250 mm × 4.6 mm, 5 μm) were used for targeted quantitative detection of evodiamine. Retention time: 8.919 min. The calibration range was 0.4 μg/mL–100 μg/mL, LOD/LOQ = 0.300, and the recovery rate was 93.29–99.80%. The precision RSD was 0.18%.

Multivariate statistical analysis was performed using orthogonal projections to latent structures–discriminant analysis (OPLS-DA) in SIMCA-P 14.1 [46]. The model was validated using goodness-of-fit (R^2^Y) and predictability (Q^2^) parameters. Metabolites with significant increases were identified based on variable importance in projection (variable importance in projection, VIP ≥ 1.0), *t*-test significance (*p* < 0.05), and fold change (FC ≥ 1.2). Correlation analysis was conducted using SPSS 26.0, and Pearson correlation coefficients (*r*-values) were calculated between the metabolite content and the relative resistance index of *L. olgensis*.

### 4.4. Transcriptomic and Proteomic Analysis of Phytoalexin Synthesis Pathways

RNA-Seq was conducted to investigate the molecular mechanisms underlying rutaevin synthesis in disease-resistant *L. olgensis*. Samples of inoculated *L. olgensis* shoots were collected, flash-frozen, and stored at −80 °C. RNA was extracted using the CTAB method, and its quality and concentration were assessed using a NanoDrop spectrophotometer (Eppendorf, BioPhotometer Model 6131). Differential gene expression (DGE) libraries were prepared and sequenced according to the Illumina/Solexa protocol, with three biological replicates per group. Gene expression levels were normalized and analyzed using FPKM (RSEM v1.2.12). Multiple hypothesis test corrections were performed according to the *P* of the tests, and the domain value of the *p* was determined by controlling the false discovery rate (FDR) [47].

The samples were sent to the BGI for protein extraction, quality control, peptide separation, mass spectrometry detection, and protein identification. Data analysis was performed using the iTRAQ quantification method [48,49]. Protein identification was further filtered using the Mascot Percolator method with a PSM-level FDR of ≤0.01 and a protein-level FDR of ≤0.01. Quantification values were then normalized, missing values were imputed, and protein quantification values were calculated and statistically analyzed (Figure S5).

Gene co-expression networks were constructed using the WGCNA package in *R* (4.1.3) [50], and modules related to rutaevin synthesis were further analyzed. Gene expression data from 8 *L. olgensis* samples (before and after *N. laricinum* inoculation) were used for network and module construction.

Gene Ontology (GO) and KEGG analyses were conducted to identify enriched biological processes and pathways. Statistical significance was set at an FDR ≤ 0.05.

### 4.5. Assessment of Fungicidal Activity of Rutaevin and Control of Larch Shoot Blight

A cross-over method was used to assess the dose-dependent inhibitory effect of rutaevin on *N. laricinum*. Rutaevin (Fifan, B28196) was dissolved in DMSO and added to PDA medium at concentrations of 1.0000 mg/mL, 0.5000 mg/mL, 0.2500 mg/mL, 0.1250 mg/mL, and 0.0625 mg/mL. The control group contained an equivalent amount of DMSO.

A 5 mm diameter mycelial disc was inoculated onto each concentration of PDA medium, with 5 replicates per treatment. Plates were incubated at 25 °C under dark conditions, and mycelial growth was measured and photographed (Canon, EOS 6D) every 24 h.

The inhibition rate (I) was calculated using the formula [51]:

(4) $$I=\frac{{\pi \left(\frac{D_{1}-0.5}{2}\right)}^{2}-{\pi \left(\frac{D_{2}-0.5}{2}\right)}^{2}}{{\pi \left(\frac{D_{1}-0.5}{2}\right)}^{2}-{\pi \left(\frac{0.5}{2}\right)}^{2}}\times 100\%$$

where *D*_1_ and *D*_2_ represent the colony diameters of the control and treatment groups, respectively. The IC_50_ was calculated based on the inhibition rate.

Rutaevin was sprayed exogenously on branch, and then the branch was inoculated with *N. laricinum*. DMSO was sprayed exogenously on the branch, and then the branch was inoculated with *N. laricinum* (control group). The inoculated group and control group were incubated in a growth chamber (25 °C, 80–90% relative humidity, 16 h of light, 8 h of darkness) and monitored until symptoms of larch shoot blight appeared. The incidence was observed, and the pathogen growth was measured, with 3 replicates in each group.

### 4.6. Validation of Antifungal Activity of Different Evodia spp. Extracts

Rutaevin in different *Evodia* spp. was extracted by methanol. The extract was added to the PDA medium, and 5 mm diameter mycelial disc was placed on the PDA medium containing the extract to identify the antifungal activity of different extracts on *N. laricinum*, with 3 replicates in each group, and the PDA medium containing methanol was used as the control. The extracts were sprayed exogenously on the branch, and then the branch was inoculated with *N. laricinum* (treated group). Methanol was sprayed exogenously on branch, and then the branch was inoculated with *N. laricinum* (control group). The treated group and control group were incubated in a growth chamber (25 °C, 80–90% relative humidity, 16 h of light, 8 h of darkness) and monitored until symptoms of larch shoot blight appeared. The antifungal effects of 3 extracts were evaluated according to the symptoms and pathogen growth. There were 3 replicates in each group.

### 4.7. Data Analysis

Time-dependent models (linear/nonlinear fitting) were applied to analyze accumulation dynamics. Fisher’s exact test and PLS regression were used to assess the relationship between gene expression and metabolite accumulation, with model performance evaluated using R^2^/Q^2^ values. Time–dose models were fitted to analyze antifungal activity and dose–response curves (AUC values). Field spraying experiments were conducted to assess disease incidence and severity index.

## 5. Conclusions

Rutaevin is a potent antifungal phytoalexin against larch shoot blight, achieving a 100% inhibition rate at a concentration of 0.5 mg/mL. And the content of rutaevin in the fruit of *E. rutaecarpa* var. *rutaecarpa* is the highest, and the extract has a good control effect against larch shoot blight. This study provides novel targets for the molecular design of disease-resistant conifers and establishes a foundation for the development of cross-disciplinary disease control strategies. The findings offer practical solutions for advancing plant disease resistance research and the development of eco-friendly fungicides, with important implications for sustainable forest disease management and ecological development.

## Data Availability

Data are contained within the article and Supplementary Materials.

## Supplementary Materials

The following supporting information can be downloaded at: https://www.mdpi.com/article/10.3390/plants14192947/s1. Figure S1. Identification of metabolites with significantly higher abundance in disease-resistant larch compared to susceptible larch. Figure S2. The content of rutaevin in disease-resistant larch and susceptible larch. Figure S3. KEGG enrichment analysis of differentially expressed genes. Figure S4. The content changes in key enzymes in disease-resistant larch after inoculation with *N. laricinum*. Figure S5. Multi-omics joint analysis flow chart. Table S1. The diameter of *N. laricinum* moss on PDA containing different concentrations of rutaevin (cm).
