# Supplementary material for: From Resistance Mechanism to Green Application: Discovery of Rutaevin as a Key Phytoalexin in Larch and Cross-Species Resource Optimization
Source: Plants (Basel). 2025 Sep 23;14(19):2947. doi: 10.3390/plants14192947 (PMC12526420; doi:10.3390/plants14192947)
Supplement: Supplementary file 1 [file plants-14-02947-s001.zip › plants-3852885-supplementary.pdf]

Supplementary Material

From Resistance Mechanism to Green Application: Discovery of Rutaevin as a Key Phytoalexin in Larch and Cross-Species Resource Optimization

Ruizhi Zhang[a, c, †], Shuang Zhang[a, †], Rui Xia[a, †], Xinyan Chen[a], Jiarui Chen[a], Feng Wang[a, b, c, *] & Danlei Li[a, c, *]

[a] Key Laboratory of Alien Forest Pest Detection and Control-Heilongjiang Province, School of Forestry, Northeast Forestry University, Harbin 150040, China

[b] State Key Laboratory of Tree Genetics and Breeding, Northeast Forestry University, Harbin 150040, China

[c] Key Laboratory of Sustainable Forest Ecosystem Management-Ministry of Education, Northeast Forestry University, Harbin 150040, China

† These authors contributed equally to this work. Ruizhi Zhang, Shuang Zhang and Rui Xia should be considered joint first author.

[*] Correspondence to: Feng Wang & Danlei Li

Feng Wang, Key Laboratory of Alien Forest Pest Detection and Control-Heilongjiang Province, School of Forestry, Northeast Forestry University, Harbin, Heilongjiang Province, China; E-mail: fengwang@nefu.edu.cn; ORCID: 0000-0002-5405-7884

Danlei Li, Key Laboratory of Alien Forest Pest Detection and Control-Heilongjiang Province, School of Forestry, Northeast Forestry University, Harbin, Heilongjiang Province, China; E-mail: danleili@nefu.edu.cn; ORCID: 0000-0003-4169-5411

1 Supplementary Figure

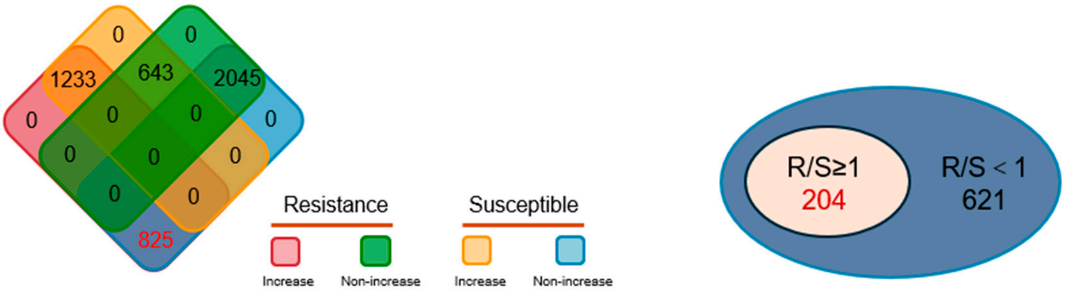

Figure S1 Identification of metabolites with significantly higher abundance in disease-resistant larch compared to susceptible larch.

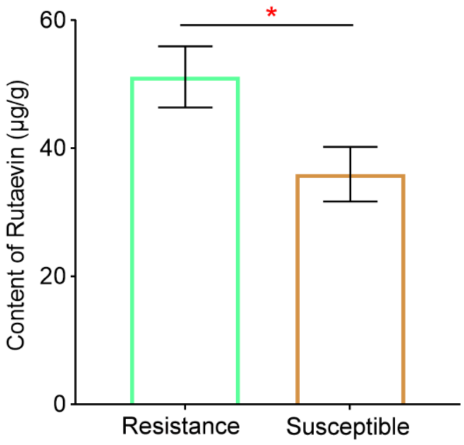

Figure S2 The content of rutaevin in disease-resistant larch and susceptible larch.

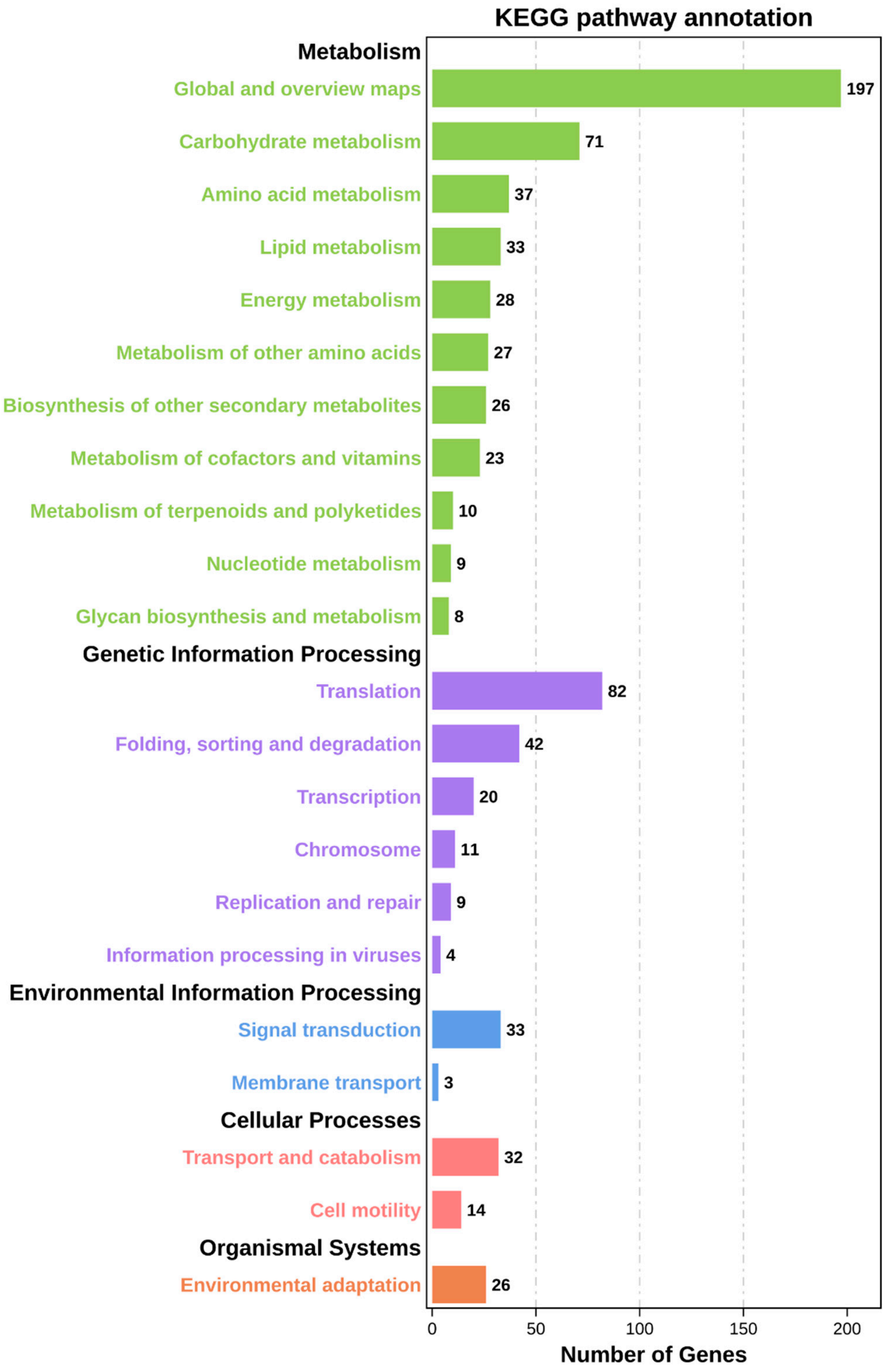

Figure S3 KEGG enrichment analysis of differentially expressed genes.

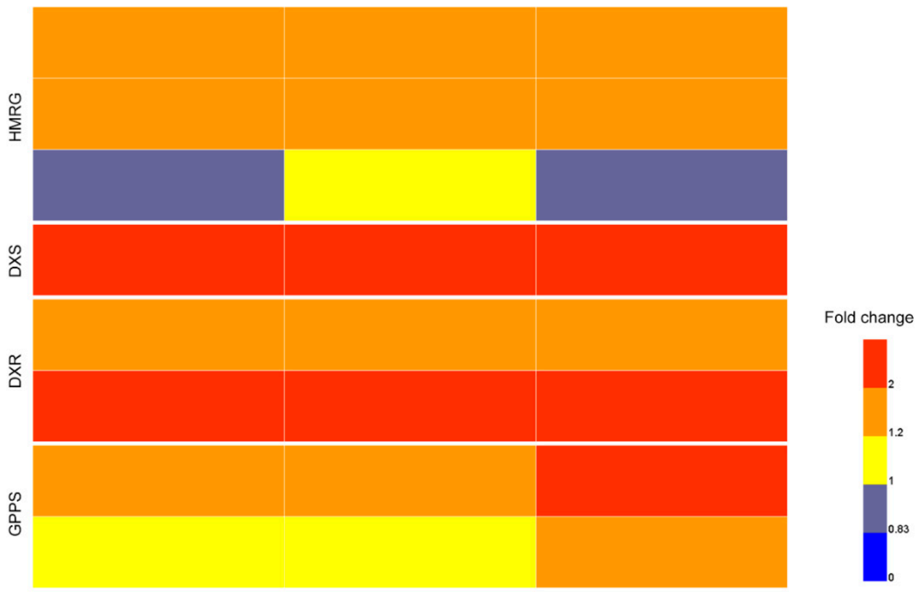

Figure S4 The content changes of key enzymes in disease-resistant larch after inoculation with *N. laricinum*.

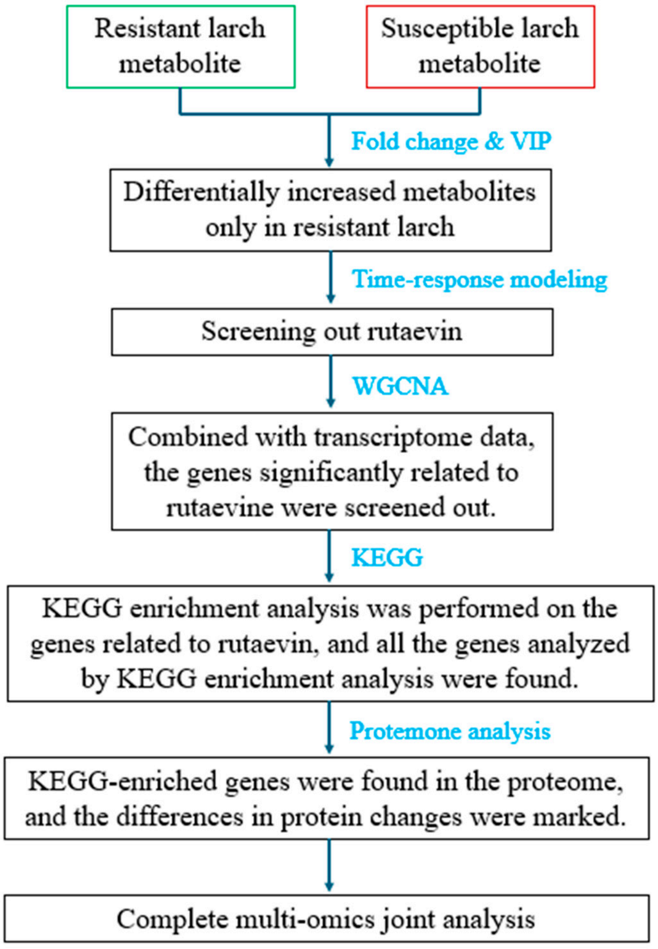

Figure S5 Multi-omics joint analysis flow chart.

Table S1 The diameter of *N. laricinum* moss on PDA containing different concentrations of rutaevin (cm).

	1 mg/mL	0.5 mg/mL	0.25 mg/mL	0.125 mg/mL	0.0625 mg/mL	CK
0 d	0.5 ±0.00	0.5 ±0.00	0.5 ±0.00	0.5 ±0.00	0.5 ±0.00	0.5 ±0.00
1 d	0.5 ±0.00	0.5 ±0.00	0.5 ±0.00	0.5 ±0.00	0.5 ±0.00	0.6 ±0.02
2 d	0.5 ±0.00	0.5 ±0.00	0.5 ±0.00	0.6±0.15	0.8±0.08	0.8±0.10
3 d	0.5 ±0.00	0.5 ±0.00	0.98±0.07	1.1±0.12	1.3±0.21	1.5±0.16
4 d	0.5 ±0.00	0.5 ±0.00	1.5±0.11	1.5±0.15	2.0±0.13	2.1±0.12
5 d	0.5 ±0.00	0.5 ±0.00	1.8±0.06	2.1±0.11	2.4±0.09	2.5±0.18
